# Notifiable Respiratory Infectious Diseases in China: A Spatial–Temporal Epidemiology Analysis

**DOI:** 10.3390/ijerph17072301

**Published:** 2020-03-29

**Authors:** Ying Mao, Rongxin He, Bin Zhu, Jinlin Liu, Ning Zhang

**Affiliations:** 1 School of Public Policy and Administration, Xi’an Jiaotong University, 28 Xianning West Road, Xi’an 710049, China; herongxin@stu.xjtu.edu.cn (R.H.); binzhu2-c@my.cityu.edu.hk (B.Z.); liujinlin_xjtu@163.com (J.L.); zhangningati@stu.xjtu.edu.cn (N.Z.); 2 Research Center for the Belt and Road Health Policy and Health Technology Assessment, Xi’an Jiaotong University, 28 Xianning West Road, Xi’an 710049, China; 3 Department of Public Policy, City University of Hong Kong, Hong Kong 999077, China; 4 Walter H. Shorenstein Asia-Pacific Research Center, Stanford University, Stanford, CA 94305, USA

**Keywords:** respiratory infectious diseases, tuberculosis, scarlet fever, measles, influenza, mumps, spatiotemporal epidemiology, Moran’s I, seasonality analysis

## Abstract

Nowadays, tuberculosis, scarlet fever, measles, influenza, and mumps are five major notifiable respiratory infectious diseases (RIDs) in China. The objective of this study was to describe, visualize, and compare the spatial-temporal distributions of these five RIDs from 2006 to 2016. In addition to descriptive epidemiology analysis, seasonality and spatial autocorrelation analysis were also applied to explore the epidemiologic trends and spatial changing patterns of the five RIDs, respectively. The results indicated that the incidence of tuberculosis, measles, and mumps presented a downtrend trend, while those of scarlet fever and influenza was in a strong uptrend across the research period. The incidences of the five diseases all peaked in spring. There were significant spatial disparities in the distribution of tuberculosis, scarlet fever, and measles cases, with the hotspots mainly located in the western plateau region, northern plain region, and southern mountainous region. To conclude, notable epidemiological differences were observed across regions, indicating that some provincial units should pay more attention to prevent and control respiratory infectious diseases.

## 1. Introduction

In 2015, the United Nations released sustainable development goals (SDGs), which proposed the elimination of the epidemics of AIDS, tuberculosis, and other infectious diseases by 2030 [1]. Respiratory infectious diseases (RIDs) refer to a number of infectious diseases involving the respiratory tract [2]. Most of them spread through droplets and short-distance contact, and the herd is generally susceptible [3]. In 2016, lower and upper respiratory tract infections accounted for 4.06% of the total disability-adjusted life years (DALYs) [4], among which lower respiratory infections caused 3.0 million deaths annually, making it the first cause of death in low-income countries [5]. In turn, tuberculosis is listed among the top 10 causes of death, with a death toll of 1.3 million [5]. As the World Health Organization (WHO) says, the control of RIDs will be a significant concern in the coming days [1].

To improve the surveillance of the epidemics of RIDs, the Chinese government increased its investment in infectious disease prevention and control systems nearly fivefold in the past decade [6]. The health professionals of the Chinese Center for Disease Control and Prevention (Chinese CDC) can record and report infections through the National Infectious Disease Direct Network Report System within several hours. After years of effort, some kinds of RIDs like pertussis, diphtheria, and meningococcal meningitis have been basically eliminated (annual incidence less than 1/100,000). However, with the continuous deterioration of air quality, the incidence of RIDs is on the rise in China, which arouses the Chinese government’s great concern. Nowadays, tuberculosis, scarlet fever, measles, influenza (three types, namely A, B, and C), and mumps are the five major notifiable RIDs in China.

Numerous researchers have explored the epidemiology of RIDs in China. Regarding tuberculosis, Wang et al. [7] performed a retrospective analysis, using the Bayesian spatiotemporal model and the spatial panel data model to investigate the spatiotemporal distribution and trends of tuberculosis prevalence in northern China from 2010 to 2014. Zhang [8] collected notification statistics of tuberculosis and imported it into a Geographic Information System, and subsequently explored the spatial–temporal epidemiological features of tuberculosis in Shandong province, China, from 2011 to 2015, and provided technical suggestions to improve future control interventions. Wang et al. [9] analyzed the spatial–temporal epidemiological characteristics of tuberculosis in Linyi City, China, from 2005 to 2010, by using spatial autocorrelation and space–time scan statistics. Nevertheless, when it comes to other RIDs, studies primarily focused on the temporal trends. Li et al. [10] reviewed the changing epidemiology of measles in Beijing, China, from 1951 to 2011. For instance, Wang et al. [11] examined measles epidemiology during 2005–2014 in Tianjin, China. Li et al. [12] described the epidemiologic characteristics of influenza cases in China. Gong et al. [13] also conducted field investigations and analyzed the epidemiological characteristics of influenza. Cui et al. [14] analyzed the epidemiological traits of mumps in China during 2008–2010.

To conclude, most of the previous research paid their attention to the temporal trends and investigation-based epidemics of RIDs, but few focused on the spatial distribution patterns and seasonality. It is no doubt that the epidemiology of RIDs differs across space, time, and season, so only a spatial–temporal analysis can reveal the RIDs’ epidemics thoroughly. Besides, few previous studies compared the epidemics of RIDs. To fill the void, this study aims to explore, visualize, and compare the spatial–temporal epidemiology of five major RIDs in China.

## 2. Materials and Methods 

### 2.1. Data Resources

Tuberculosis, scarlet fever, measles, influenza, and mumps are all notifiable infectious diseases under the strict monitoring of China’s CDC. We collected the provincial year-end and month-end incidence data in all the available years (2006–2016) from the public health science data center of China’s CDC. We obtained the population data from the National Bureau of Statistics (NBS). The research reports on the panel data of RIDs at the 31 provincial units in China, excluding Hong Kong, Macau, and Taiwan, due to data accessibility. Table S1 shows the incidence of RIDs in each provincial unit during 2006–2016. Table S2 shows the number of cases and the incidence of RIDs by month.

### 2.2. Time-Series Analysis 

The time-series analysis indicated the temporal trends of RIDs incidence (new cases per 100,000 people) in each province and also across the nation. In the first place, the box plots are used to display the statistics (median, minimum, upper quartile, lower quartile, and outliers), and display the overall temporal trends of the incidence of RIDs. Secondly, the growth rates of the incidence in each provincial unit during the study period (2006–2016) are exhibited, and units that displayed significant linear trends were identified by the chi-square linear-by-linear association test.

### 2.3. Seasonality Analysis

Affected by the seasonal factors, RIDs have obvious seasonality [15,16]. Seasonality analysis indicated the seasonal characteristics of RIDs. On the one hand, the seasonal decomposition deconstructs a time series into some kinds of components, which represents one of the underlying patterns [17,18]. This research decomposition based on RIDs incidence of change breaks the time series of RIDs into:

Zt = Tt + St + Rt.

All these component series have a certain characteristic or type of behavior [19,20,21]. Zt represents the monthly incidence of the diseases. Tt represents a trend component, which reflects the long-term progression and repeated but non-periodic fluctuations of the series. A trend exists when there is a persistent increasing or decreasing direction in the data. The trend component does not have to be linear. St represents the seasonal component, reflecting seasonality, which occurs over a fixed and known period. A seasonal pattern exists when a time series is influenced by seasonal factors. Seasonality occurs over a fixed and known period. Rt represents the remainder component, which describes random, irregular influences. It represents the residuals or remainder of the time series after the other components have been removed. For this research, we used a seasonal scan statistic, which ignores the observation of the whole year and only cares about the day and month to detect the seasonal cluster of RIDs [22].

### 2.4. Spatial Autocorrelation Analysis 

Moran’s I is an important indicator to analyze the spatial distribution characteristics of disease cases, and has been used in a lot of studies [23,24,25]. It has two forms: the global and local Moran’s I (formula in the Appendix A). The global Moran’s I was used to identify spatial autocorrelation and detect the spatial distribution pattern of RIDs in China. Moreover, the local level of spatial autocorrelation and the locations of clusters were examined by the local Moran’s I. With the constitution of the border-based spatial weight matrix, Moran’s I statistics quantify the spatial clustering patterns between neighboring units. 

The value of Moran’s I ranges from -1 to 1, where a 0 indicates that the RIDs cases are randomly distributed in space and no clusters are detected [26]. A value approaching 1 indicates the unit clusters with a similar value [27]. A value approximating -1 indicates an opposing situation: the units with high values and low values are adjacent to each other in space. The local Moran’s I detect the clusters based on the administrative divisions. Based on its value and significance, the local Moran’s I can detect four types of clusters, reflecting the high–high (HH, geographical units with high value surrounded by geographical units with low value), high–low (HL, geographical units with high value surrounded by geographical units with high value), low–low (LL), and low–high (LH) clustering patterns, respectively [28]. The local Moran’s I is a kind of Local Indicator of Spatial Association (LISA); thus, the maps that display the clusters detected by the local Moran’s I are always termed as univariate LISA cluster maps [27]. The number of permutation tests was set to 9999, and the significance level was set as 0.05.

### 2.5. Software Tools

The values of Moran’s I were calculated by using the software GeoDa 1.8.61 (the University of Chicago, Chicago, IL, USA). The box plots were drawn with Microsoft Excel 2016 (Microsoft Corp., Redmond, WA, USA). The chi-square linear-by-linear association tests and the seasonal decomposition were conducted in SPSS 20.0 (IBM Inc., Armonk, NY, USA). The seasonal scan statistic was measured with SaTScan 9.5 (Kulldorff and Information Management Services, Inc., Boston, MA, USA). All the maps were drawn and visualized in ArcGIS 10.0 (ESRI Inc., Redlands, CA, USA).

## 3. Results

### 3.1. Epidemiologic Trends

**Figure 1:**
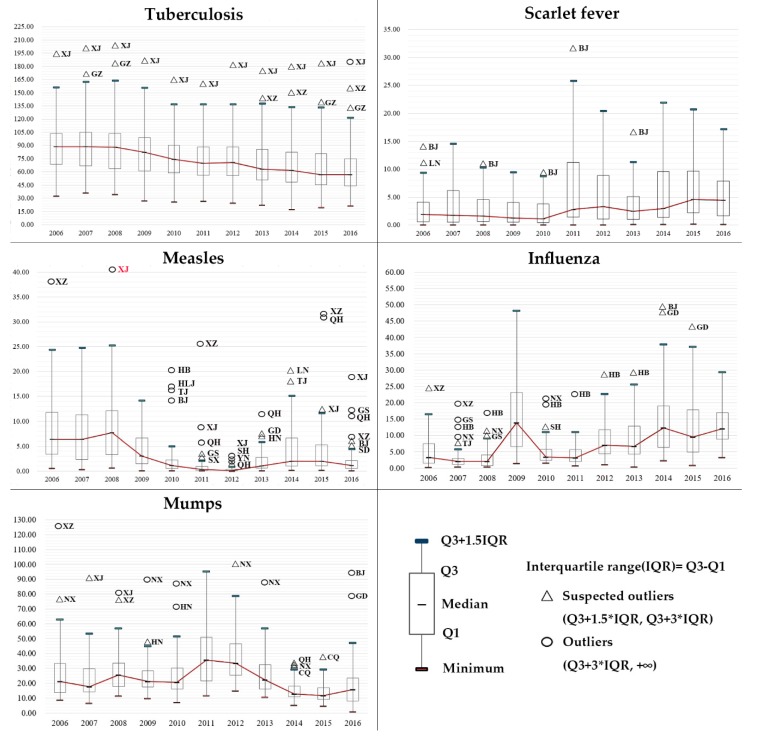
Box plots of the incidence of different types of respiratory infectious diseases (RIDs) (2006–2016).

Figure 1 displays the temporal trends of five kinds of RIDs in boxplots. The suspected outliers and outliers were identified and marked on the box plots, displaying the incidence of RIDs of some provincial units that do not fit the rest of the data. Each type of RID displayed distinct epidemiologic trends. The upper quartile, median, and lower quartile of the incidence of tuberculosis gradually decreased since 2006, while Xinjiang was identified as a suspected outlier, and turned into an outlier in 2016. As for scarlet fever, its upper quartile, median, and lower quartile remained relatively stable in 2006–2010, but began to increase rapidly since 2011. Beijing was identified as a suspected outlier in some years. The temporal trend of measles was opposite: the median, upper quartile, lower quartile, and IQR (interquartile range, the difference between the upper and lower quartile) all decreased dramatically during 2009–2012, but in 2013 they were showing a growing trend again. The outliers were mainly distributed in west China. In contrast, the median for the incidence of influenza gradually increased since 2006, except for a particularly high increase in 2009. The outliers and suspected outliers are mainly in densely-populated areas, such as Shanghai, Beijing, Guangdong, and Hebei. Different from the temporal trends described above, the median for the incidence of mumps presented a fluctuating trend, with western China showing a relatively high incidence of mumps across the whole research period. Overall, the incidence trend of tuberculosis, measles, and mumps presented a downtrend trend, whereas the incidence of scarlet fever and influenza have been in a strong upward trend from 2006 to 2016.

To better understand the temporal trends of RIDs in China, we also compute the growth rate of the incidence of RIDs in each provincial unit in Table 1, in which the incidence and growth rate across 2006 to 2016 can be seen. In most provincial units, the incidence trend was consistent with the general tendency, while the growth rates of RIDs in some provincial units differed considerably and even displayed a reverse trend. From 2006 to 2016, the incidence of tuberculosis in the Xizang and Qinghai provinces increased by 5.41% and 5.52%, respectively, which was in sharp comparison to the −3.34% among other units. Similarly, the incidence of measles in the Xinjiang, Gansu, Qinghai, and Ningxia provinces were opposite to the general tendency (−13.34%). The provinces mentioned above all are mainly distributed in western China. The incidence of mumps in some central and eastern provinces of China (Hebei, Shanxi, Anhui, Fujian, Jiangxi, Henan, Hunan, Hubei, and Guangdong) also presented an opposite trend.

### 3.2. Seasonal Decomposition

**Figure 2:**
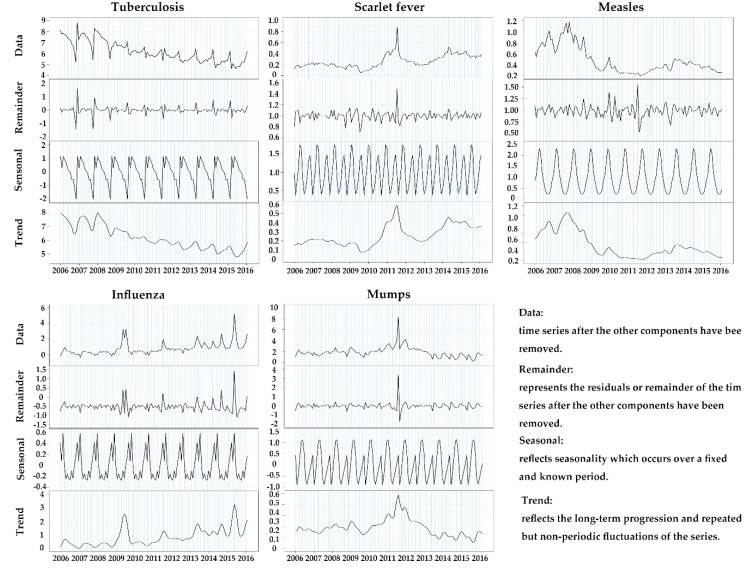
Seasonal decomposition of the monthly incidence of reportable RIDs (2006–2016) in China.

The seasonal decomposition of the reportable RIDs rates is displayed in Figure 2. The incidence of tuberculosis peaked in late winter and early spring. The incidence of scarlet fever showed a bimodal seasonal pattern with the more massive peaks occurring in the summer and a smaller peak occurring in winter. The incidence of measles peaked in spring. A bimodal seasonal pattern was also observed in the seasonal decomposition of the incidence of influenza. Two towering peaks were observed in winter and spring. A smaller peak can be seen in late autumn. The seasonal decomposition of the incidence of mumps indicated a bimodal seasonal pattern with the peak incidence occurring during the summer months with a peak of about half the size occurring in spring.

### 3.3. Seasonal Scan Statistic

The temporal scan statistic identified temporal clusters during the study period (2006–2016), presented in Table 2. We found statistically significant (*p* < 0.01) different infection peaks for each disease. In Supplementary Table S3, the months highlighted indicate the peaks in each year. The temporal clusters that were identified for the incidence of tuberculosis occurred between January and May. For the incidence of scarlet fever infections, temporal clusters all occurred in the summer months. Temporal clusters of the incidence for measles occurred in late winter, extending into the early summer months. For influenza, there is a wide-ranging time frame. Temporal clusters came up in late autumn and extended into the spring months. Temporal clusters of mumps occurred in spring and extended into the summer months. The results confirm the conclusion of the seasonal decomposition we mentioned above.

### 3.4. Global Spatial Autocorrelation

The global spatial autocorrelation of RIDs and their test results are displayed in Table 3. For tuberculosis, the global Moran’s I reached up to the significance level of 0.05 in most years. The global Moran’s I for scarlet fever all reached the significance level of 0.01 during the study period, indicating a robust spatial cluster tendency of the reported cases. In contrast, the global Moran’s I for measles displayed a drastic fluctuation. It was higher than 0.3 from 2014 to 2016. Similarly, the global Moran’s I for influenza and mumps went through a marked change, presenting a spatial cluster tendency in some years.

### 3.5. Local Spatial Autocorrelation

**Figure 3:**
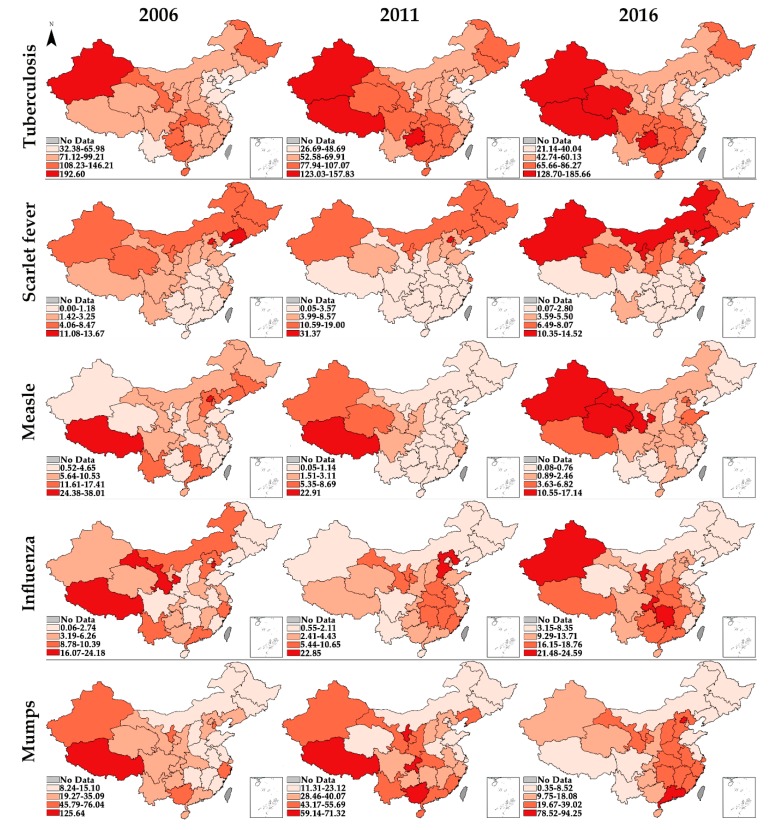
Hierarchy maps of the incidence of RIDs in 2006, 2011, and 2016.

As the local spatial autocorrelation analysis only reveals the relative state rather than the absolute incidence in each provincial unit, we divided all the 31 provincial units in China into four classes in hierarchy maps (Figure 3) based on the maximum and minimum of one specific type of RID during 2006, 2011, and 2016. For example, the maximum and minimum incidence of tuberculosis in 2006 are 192.60 and 32.38, respectively. Then the difference between these two values was divided evenly into four levels. The darker the red color, the higher the incidence of RIDs.

**Figure 4:**
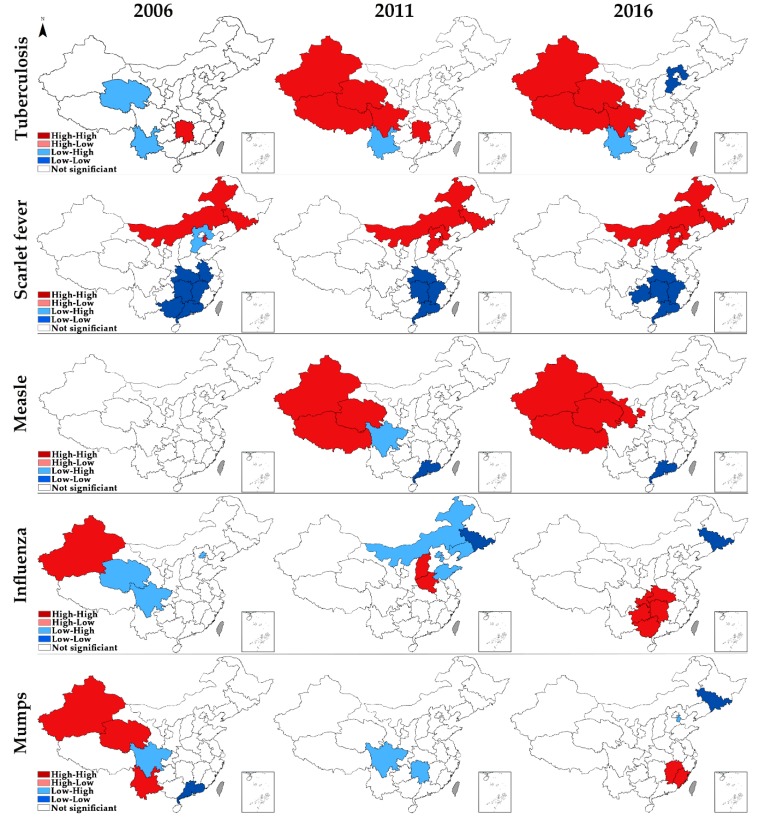
Spatial clusters of RIDs in 2006, 2011, and 2016.

Figure 4 shows the spatial clusters for RIDs in 2006, 2011, and 2016, which reveals the spatial variation patterns of RIDs in China. Only those units whose local Moran’s I have reached the significance level of 0.05 were presented on the maps. For tuberculosis in 2006, the HH cluster was located in Hunan, central China, while Qinghai and Yunnan displayed a LH cluster feature. During the first sub-period (2006–2011), Qinghai moved out of the LH cluster area, while most parts of western China began to display the HH cluster feature. When it comes to 2016, Hunan departed from the HH cluster area, and Hebei began to present the LL cluster feature. 

Regarding scarlet fever, the HH cluster area remained in north China, and the LL area remained in south China across the study period. From 2006 to 2011, Hebei also began to display the HH cluster feature, and the HH cluster area thus extended. Anhui and Guangxi moved out of the LH cluster area, and the LL cluster area thus shrunk. Moreover, Guizhou moved into the LL cluster area in 2016. 

As for measles, no provincial units were displaying any cluster feature in 2006. During the first sub-period, the HH cluster area appeared in western China, and the HH cluster area thus extended in 2016. Sichuan began to display the LH cluster feature and moved out in 2016. Guangdong remained relatively stable after 2011.

In terms of influenza, Xinjiang displayed the HH cluster feature, and the LH cluster was located in Qinghai, Sichuan, and Beijing in 2006. When the time came to 2011, the HH cluster area gradually moved into central China, and the LH cluster area gradually moved into northeast China. Besides, Jilin presented the LL cluster feature. From 2011 to 2016, the LH cluster area wears off, and the HH cluster area gradually moved southward. Jilin remained the LL cluster feature. 

As for mumps, the HH cluster was located in a part of western China, whereas Sichuan and Guangdong displayed the LH and LL cluster features, respectively, in 2006. From 2006 to 2011, the HH and LL cluster areas wear off. Hunan moved into the LH cluster area. From 2011 to 2016, the HH cluster area of mumps moved southeastward. The LH cluster area moved northward, while Jilin displayed the LL cluster feature. 

## 4. Discussion

This study revealed the spatial–temporal epidemiology of five major RIDs (tuberculosis, scarlet fever, measles, influenza, and mumps) in China, which provided insight into potential solutions to diminish the diseases in China. We want to discuss the spatial–temporal epidemiology of these five major RIDs first, and then explore future prevention and control strategies.

In the first place, we compared the temporal trends of the five RIDs. Based on the incidence of RIDs between 2006 and 2016, their temporal trends had similarities and differences. Regarding the number of cases, the incidence of tuberculosis was still far above that of other RIDs, despite it having been on a declining curve from 2006 to 2016, which is consistent with the results from the studies conducted by Cao et al. [29] and Guo et al. [30]. The hotspots, high-risk groups, and high-occurrence seasons of tuberculosis still need special attention from all stakeholders. Then, based on the incidence recorded above, influenza and mumps were less severe diseases. Scarlet fever and measles were the least severe diseases. Regarding the trend, the incidence of influenza was ascending notably, with the outbreak of influenza A (H1N1) in 2009 [31]. Scarlet fever resurged since 2011, in line with the results from the study by Liu et al. [32], which explained that the upsurge in scarlet fever in China was a natural cyclical pattern of scarlet fever. A report [33] has indicated that the gene of scarlet fever had highly diversified clones with a high macrolide resistance that causes the re-emergence. This phenomenon deserves the vigilance of the health sector. Then, the incidence of measles has dropped sharply since 2008 and reached its lowest level in 2012 due to a 6-year measles elimination campaign in China, which rapidly narrowed the immunity gap in children by >95% coverage of a 2-dose routine measles-containing vaccine (MCV) [34]. Even the epidemic of tuberculosis was controlled, but drug-resistant tuberculosis still remains a severe epidemic, which is associated with inadequate treatment in both the public health system and the medical service system in China [35]. The trend in the incidence of mumps was fluctuant in the study period. There was an epidemic during 2011–2012, even after a mumps-containing vaccine (MuCV) was formally introduced into the national immunization program in China in 2008. Cui et al. [14] reported it might result from the accumulation of many mumps-susceptible individuals and low mumps vaccination coverage. After that, the incidence of mumps significantly decreased.

Secondly, regarding the seasonal changes, the incidence of tuberculosis and influenza both peaked in late winter and early spring. Scarlet fever showed a bimodal seasonal pattern with the larger peaks occurring in the summer and a smaller peak occurring in winter. Measles displayed a high-incidence season in spring, and mumps peak incidence occurred during the summer months with a peak of about half the size occurring in spring. Previous studies have revealed an association between the RIDs and seasons [18,22,30,36,37]. The occurrence of RIDs is related to climatic factors, such as the amount of sunshine, changes in the temperature, and indoor activity. High temperature and humidity are ideal for the rapid growth of bacteria. Furthermore, a complex relationship can be speculated among factors, for instance, an increase in indoor activities during winter and spring, leading to crowded living, which brings about bad ventilation and increases the chance of infection. So, the health sector and the education sector need to cooperate to change the lifestyle of residents and improve their self-protection awareness. In conclusion, during late winter to the next summer is the season when the incidence of RIDs peaked and need special attention. The health sector should strengthen the surveillance and knowledge publicity of the corresponding RIDs during these months.

Thirdly, the high-risk areas for different RIDs were mainly located in the western plateau region, northern plain region, and southern mountainous region of China. The western plateau region is the hotspot for tuberculosis and measles. Most of the western plateau regions are remote areas with high altitude, frigid climate, where the cold, dry weather may cause high herd susceptibility of tuberculosis and measles [17,34,38]. Furthermore, this region is combined with several undeveloped provinces, which are in critical lack of health resources, leading to delays in detection and diagnosis, as well as to low vaccine coverage of infectious diseases. Furthermore, some studies also found that residents who lived in this region lack self-protection awareness [39,40]. So, in this region, the health sector should maintain sufficient health resources supply and improve residents’ health literacy. The northern plain region is the high-risk area for scarlet fever, and the south is the opposite. Site-to-site differences in risk factors might have remained, such as long-term exposure to dangerous air pollutants in the northern regions, which could have been a risk factor for infection [41]. The health sector and environment sector should collaborate and formulate some joint measures to alleviate the impact of air pollution on the incidence of infectious diseases. Besides, the epidemiological surveillance indicated that the proportion of group A streptococcus emm12 gene type strains was gradually replaced by the emm1 gene types in some areas in the north of China from 2011 to 2014, leading to minimal cross-immunity [34]. For influenza and mumps, the results of global spatial autocorrelation were not significant in some years, indicating influenza and mumps was prevalent in all the provincial units. However, from a regional perspective, the high-risk areas of influenza and mumps noticeably moved southward from 2006 to 2016 due to the spread of vaccines in western China [14]. Now, the southern mountainous region in China has become the severely afflicted area, which has a subtropical and humid climate and is characterized by high temperatures, precipitation, and humidity. Moreover, the rapid growth of population density and a large migrant population in this region also has contributed to the trend [42]. The health sector should pay special attention to RIDs control of the mobile population. Once an outbreak of RIDs is detected, the population mobility should be restricted immediately. More importantly, every provincial unit should make an evidence-based solution that identifies the priority measures. For those adjacent provincial units displaying HH or HL cluster features, it could be more efficient if they can collaborate with each other, thereby establishing interconnected networks for the sharing of information.

There are some limitations to this study. We only did the analysis at the provincial level as we were unable to obtain more detailed statistics for the municipal units or county units. Studies that explore the temporal and spatial distributions in smaller region units are suggested for the future.

## 5. Conclusions

In conclusion, trends, seasonal patterns, and spatial disparities in different provincial geographical units were determined. Different RIDs display different trends. The incidence of influenza was notably ascending, while that of scarlet fever indicated a resurged trend; the epidemic of tuberculosis, measles, and mumps was under control. Higher incidences of RIDs were observed from January to June, and the climate zone is associated with this high incidence. The high-risk areas for RIDs were detected. Each high-risk region faces different challenges as there exist distinct risk factors due to the different natural environments and economic development, so every provincial unit should create an evidence-based solution that identifies the priority measures and enables meaningful inputs from all key stakeholders. In particular, the measures to deal with respiratory infectious diseases in China should not be restricted to its prevention and control.

## Figures and Tables

**Table 1:** The growth in incidence of RIDs in China and the linear test (1/100,000) ^1^.

Region	Tuberculosis			Scarlet Fever			Measles			Influenza			Mumps		
	2006	2016	Growth	2006	2016	Growth	2006	2016	Growth	2006	2016	Growth	2006	2016	Growth
Beijing	51.29	31.01	−4.91% ***	13.67	12.08	−1.23%	24.38	5.75	−13.45% *	1.89	10.09	18.27%	28.21	94.25	12.82%
Tianjin	36.54	21.14	−5.33% ***	4.06	11.21	10.70% **	13.02	3.63	−11.99%	16.07	9.43	−5.19%	52.39	15.74	−11.33% ***
Hebei	62.54	45.34	−3.16% ***	2.64	5.21	7.04% **	11.61	2.46	−14.37%	10.39	9.97	−0.41% *	21.00	39.02	6.39%
Shanxi	71.12	38.65	−5.92% ***	2.49	7.69	11.94% **	6.71	0.41	−24.38% **	2.15	10.23	16.88% **	10.94	20.52	6.48%
Neimenggu	90.05	48.30	−6.04% ***	6.80	10.35	4.29% **	10.53	1.12	−20.07%	9.60	7.37	−2.62%	12.82	8.52	−4.00%
Liaoning	56.20	51.40	−0.89%	11.08	10.36	−0.67%	12.82	0.26	−32.28%	0.21	6.87	41.67% ***	24.54	4.61	−15.39% *
Jilin	83.56	49.76	−5.05% ***	4.46	7.68	5.58%	17.41	0.31	−33.16%	0.18	4.07	36.30% ***	11.87	3.19	−12.32%
Heilongjiang	109.35	80.16	−3.06% ***	8.47	7.43	−1.31%	8.97	0.29	−29.05%	0.06	3.15	47.90%	15.10	2.74	−15.68% **
Shanghai	32.38	27.28	−1.70% ***	3.25	12.10	14.05% **	3.36	0.69	−14.65% *	0.43	9.81	36.62% **	21.33	19.67	−0.81% **
Jiangsu	65.99	35.93	−5.90% ***	1.18	2.80	9.04% **	6.10	0.94	−17.06% **	6.23	6.33	0.16%	12.48	6.56	−6.23%
Zhejiang	88.74	48.78	−5.81% ***	1.90	4.46	8.91% **	3.21	0.59	−15.57%	8.78	12.77	3.82% *	46.94	26.13	−5.69% ***
Anhui	85.63	56.77	−4.03% ***	0.30	1.33	16.10% **	2.45	1.17	−7.13%	2.34	12.69	18.43% ***	14.31	23.76	5.20%
Fujian	85.87	42.74	−6.74% ***	0.36	1.59	16.04% ***	4.65	0.63	−18.12% **	1.74	7.72	16.09% **	14.52	27.15	6.46%
Jiangxi	98.49	71.99	−3.09% ***	0.05	0.10	7.98% **	3.68	0.50	−18.10% ***	4.73	16.75	13.48% ***	8.66	20.83	9.18%
Shandong	42.24	30.78	−3.12% ***	1.43	7.22	17.56% ***	3.50	4.47	2.46%	0.18	6.50	43.44% ***	8.24	7.34	−1.14%
Henan	95.98	60.13	−4.57% ***	0.66	1.56	9.04% **	7.78	1.19	−17.12% **	3.19	16.80	18.08% ***	9.13	23.47	9.90% *
Hubei	108.23	74.70	−3.64% ***	0.40	1.73	15.69% ***	4.02	1.21	−11.31% **	2.74	17.89	20.63%	19.27	19.96	0.35%
Hunan	91.99	75.50	−1.96% ***	0.17	1.15	20.90% ***	11.99	1.16	−20.83% **	1.41	23.41	32.41%	13.35	23.56	5.85%
Guangdong	95.22	71.82	−2.78% ***	0.37	2.40	20.42% ***	15.10	1.17	−22.57% **	8.78	16.24	6.35% **	20.45	78.52	14.40%
Guangxi	127.23	86.27	−3.81% ***	0.46	1.09	8.93% **	1.90	0.08	−27.15% **	4.29	18.76	15.90%	45.79	18.08	−8.87%
Hainan	140.54	84.18	−5.00% ***	0.00	0.07	−	9.23	1.76	−15.27%	0.58	13.71	37.21% ***	21.11	11.59	−5.82%
Chongqing	124.67	73.34	−5.17% ***	1.42	1.60	1.21% *	5.64	1.13	−14.86%	4.15	24.41	19.39%	23.30	10.54	−7.63%
Sichuan	99.21	65.66	−4.04% ***	1.73	2.10	1.96% **	9.95	0.99	−20.61% *	2.50	10.69	15.63%	23.73	4.90	−14.59% **
Guizhou	146.21	130.66	−1.12% **	0.81	2.05	9.74% ***	1.01	0.09	−21.50% *	4.73	17.54	14.00%	32.67	9.75	−11.39% **
Yunnan	60.42	55.47	−0.85% ***	1.75	3.59	7.46% **	14.47	0.34	−31.28% **	10.08	12.02	1.77%	35.09	5.63	−16.71% *
Xizang	91.17	154.37	5.41% ***	2.89	2.44	−1.67%	38.01	6.82	−15.78%	24.18	17.19	−3.35%	125.64	0.35	−44.53% **
Shaanxi	87.12	56.30	−4.27% ***	2.43	8.07	12.76% **	2.70	0.89	−10.49% **	0.95	16.15	32.73% **	21.08	15.83	−2.83%
Gansu	108.23	58.13	−6.03% ***	2.86	5.50	6.76% **	6.39	11.14	5.72%	16.24	9.29	−5.43%	34.06	32.73	−0.40%
Qinghai	75.21	128.70	5.52% ***	6.10	6.49	0.63%	3.44	10.55	11.85% **	3.26	8.35	9.86% **	32.12	13.15	−8.55% **
Ningxia	76.86	40.04	−6.31% ***	4.16	14.52	13.31% ***	0.52	0.76	3.87%	5.29	21.48	15.05%	76.04	21.68	−11.79%
Xinjiang	192.60	185.66	−0.37%	4.70	12.69	10.45% **	1.49	17.14	27.69%	6.26	24.59	14.67%	47.93	10.64	−13.97% **
SUM	86.23	61.38	−3.34% ***	2.11	4.35	7.50% **	7.62	1.82	−13.34% **	4.40	22.51	17.73% **	20.76	12.84	−4.69%

^1^ Growth rates in parentheses; units that displayed a significant linear trend during the subperiod are in bold. * Statistical significance at the 10% level; ** Statistical significance at the 5% level; *** Statistical significance at the 1% level.

**Table 2:** The temporal clusters of reportable RIDs (2006–2016) in China.

Infections	Time Frame(Months)	Number of Cases	Expected Cases	Observed/Expected	Relative Risk	Log-Likelihood Ratio	*p*-Value
Tuberculosis	January to May	6,709,673	6,047,427.81	1.11	1.20	61,857.01	0.001
Scarlet fever	May to June	128,684	77,291.75	1.66	1.92	17,795.86	0.001
Measles	February to June	450,685	264,491.11	1.70	3.35	109,997.33	0.001
Influenza	November to April	893,826	711,198.12	1.26	1.67	46,159.38	0.001
Mumps	April to July	1,559,324	1,108,119.59	1.41	1.77	131,010.74	0.001

**Table 3:** Global spatial autocorrelation analysis and test results.

Year	Tuberculosis			Scarlet Fever			Measles			Influenza			Mumps		
	Moran’s I	Z−Value	*p*−Value	Moran’s I	Z−Value	*p*−Value	Moran’s I	Z−Value	*p*−Value	Moran’s I	Z−Value	*p*−Value	Moran’s I	Z−Value	*p*−Value
2006	0.108	1.2335	0.1109	0.3783	3.8401	0.0018	0.034	0.6079	0.25	−0.0647	−0.3374	0.3898	0.172	2.0203	0.0327
2007	0.1334	1.4341	0.0815	0.4563	4.4060	0.0002	−0.2396	−1.8489	0.0220	−0.0636	−0.3443	0.4021	0.3355	3.5332	0.0016
2008	0.1001	1.1422	0.1260	0.4767	4.5124	0.0002	−0.0434	−0.2322	0.4298	−0.0354	−0.0731	0.4922	0.2268	2.3393	0.0182
2009	0.1162	1.2921	0.0968	0.4454	4.2746	0.0002	0.1162	1.2789	0.1055	0.2025	2.0695	0.0274	0.0090	0.3644	0.3368
2010	0.1266	1.3756	0.0852	0.4858	4.6689	0.0002	0.4776	4.8974	0.0011	−0.0502	−0.2139	0.4518	−0.0082	0.1913	0.3925
2011	0.2266	2.2525	0.0161	0.3984	3.9892	0.0008	0.2995	4.7804	0.0032	−0.0621	−0.3611	0.3735	0.0663	0.8351	0.1985
2012	0.2844	2.8054	0.0053	0.4254	4.0517	0.0003	−0.0777	−0.4547	0.3509	0.0439	0.6526	0.2422	0.1488	1.6101	0.0597
2013	0.3171	3.0903	0.0031	0.3028	3.1607	0.0004	−0.0840	−0.5553	0.2982	0.0349	0.5764	0.2686	0.1776	1.9877	0.0309
2014	0.2732	2.7102	0.0073	0.4329	4.0828	0.0004	0.3177	3.1262	0.0053	0.0123	0.3777	0.3255	0.1060	1.2057	0.1149
2015	0.3114	3.0845	0.0034	0.3723	3.2113	0.0039	0.3643	4.1316	0.0066	−0.0721	−0.4012	0.3584	0.1231	1.3948	0.0859
2016	0.3497	3.4609	0.0017	0.3761	3.5555	0.0006	0.5228	5.5626	0.0004	0.1991	2.0050	0.0295	−0.0454	−0.1709	0.4639

## Supplementary Materials

The following are available online at https://www.mdpi.com/1660-4601/17/7/2301/s1, Table S1: The incidence of RIDs in each provincial unit during 2006–2016, Table S2: the number of cases and the incidence of RIDs by month. Table S3: Highlighted cells represent the months of infection peaks.

## Abbreviations

Chinese CDC	Chinese Center for Disease Control and Prevention
DALYs	Disability-Adjusted Life Years
HH	High–High
HL	High–Low
LH	Low–High
LISA	Local Indicator of Spatial Association
LL	Low–Low
MCV	Measles-Containing Vaccine
MuCV	Mumps-Containing Vaccine
NBS	National Bureau of Statistics
RIDs	Respiratory Infectious Diseases
SDGs	Sustainable Development Goals

## Appendix A

Moran’s I:

(A1) $${\mathrm{Global}\;\mathrm{Moran}}^{\prime }I=\frac{n\sum_{i=1}^{n}\sum_{j=1}^{n}SW_{ij}\left(y_{i}-\bar{y}\right)\left(y_{j}-\bar{y}\right)}{\left(\sum_{i=1}^{n}\sum_{j=1}^{n}SW_{ij}\right)\sum_{i=1}^{n}{\left(y_{i}-\bar{y}\right)}^{2}}$$

(A2) $${\mathrm{Local}\;\mathrm{Moran}}^{\prime }I=\frac{\left(y_{i}-\bar{y}\right)}{u_{0}}\sum_{j}SW_{ij}\left(y_{j}-\bar{y}\right)\;\;u_{0}=\sum_{j}{\left(y_{i}-\bar{y}\right)}^{2}/n$$

n: the number of geographical units (31 provincial units in this study).
$y_{i}$: the incidence of one certain type of RIDs in geographical unit i.
$y_{j}$: the incidence of one certain type of RIDs in geographical unit j.
$\bar{y}$: the average incidence of one certain type of RIDs in all the geographical units.
$SW_{ij}:\;$spatial-weighted n×n matrix which represents neighboring relations. $SW_{ij}$= 1 if unit i is adjacent with unit j, and $SW_{ij}$ = 0 otherwise.

## References

[1] WHO (2016). World Health Statistics: Monitoring Health for the SDGs, Sustainable Development Goals.

[2] Vink M.A., Christoffel M., Bootsma J., Wallinga J. (2014). Systematic reviews and meta- and pooled analyses serial intervals of respiratory infectious diseases: A systematic review and analysis. Am. J. Epidemiol..

[3] Sheffield E.R.S. (2017). The Global Impact of Respiratory Disease.

[4] Wang H., Abajobir A.A., Abate K.H., Abbafati C., Abbas K.M., Abd-Allah F., Abera S.F., Abraha H.N., Abu-Raddad L.J., Abu-Rmeileh N.M.E. (2017). Global, regional, and national under-5 mortality, adult mortality, age-specific mortality, and life expectancy, 1970-2016: A systematic analysis for the Global Burden of Disease Study 2016. Lancet.

[5] Region E. (2018). Progress towards the SDGs: A Selection of Data from World Health Statistics 2018 SDG3: Ensure Healthy Lives and Promote Well-Being for all Ages.

[6] Li C., Sun M., Wang Y., Luo L., Yu M., Zhang Y., Wang H., Shi P., Chen Z., Wang J. (2016). The centers for disease control and prevention system in China: Trends from 2002-2012. Am. J. Public Health.

[7] Wang X., Yin S., Li Y., Wang W., Du M., Guo W., Xue M., Wu J., Liang D., Wang R. (2019). Spatiotemporal epidemiology of, and factors associated with, the tuberculosis prevalence in northern China, 2010–2014. BMC Infect. Dis..

[8] Zhang X. (2017). Spatial-temporal epidemiological characteristics of tuberculosis in Shandong Province, China in 2011–2015. EC Pulmonol. Respir. Med..

[9] Wang T., Xue F., Chen Y., Ma Y., Liu Y. (2012). The spatial epidemiology of tuberculosis in Linyi, China, 2005–2010. BMC Public Health.

[10] Li J., Lu L., Pang X., Sun M., Ma R., Liu D., Wu J. (2013). A 60-year review on the changing epidemiology of measles in capital Beijing, China, 1951–2011. BMC Public Health.

[11] Wagner A.L., Boulton M.L., Gillespie B.W., Zhang Y., Ding Y., Carlson B.F., Luo X., Montgomery J.L.P., Wang X. (2017). Risk factors for measles among adults in Tianjin, China: Who should be controls in a case-control study?. PLoS ONE.

[12] Li Q., Zhou L., Zhou M., Chen Z., Li F., Wu H., Xiang N., Chen E., Tang F., Wang D. (2014). Epidemiology of human infections with avian influenza A(H7N9) virus in China. N. Engl. J. Med..

[13] Gong Z., Lv H., Ding H., Han J., Sun J., Chai C., Cai J., Yu Z., Chen E. (2014). Epidemiology of the avian influenza A (H7N9) outbreak in Zhejiang Province, China. BMC Infect. Dis..

[14] Cui A., Zhu Z., Hu Y., Deng X., Sun Z., Zhang Y., Mao N., Xu S., Fang X., Gao H. (2017). Mumps epidemiology and mumps virus genotypes circulating in mainland China during 2013–2015. PLoS ONE.

[15] Khor C., Sam I., Hooi P., Quek K., Chan Y. (2012). Epidemiology and seasonality of respiratory viral infections in hospitalized children in Kuala Lumpur, Malaysia: A retrospective study of 27 years. BMC Pediatr..

[16] Walsh M.G., Amstislavski P., Greene A., Haseeb M.A. (2017). The landscape epidemiology of seasonal clustering of highly pathogenic avian influenza (H5N1) in domestic poultry in Africa, Europe and Asia. Transbound. Emerg. Dis..

[17] Khaliq A., Batool S.A., Chaudhry M.N. (2015). Seasonality and trend analysis of tuberculosis in Lahore, Pakistan from 2006 to 2013. J. Epidemiol. Glob. Health.

[18] Wang R., Jiang Y., Guo X., Wu Y., Zhao G. (2018). Influence of infectious disease seasonality on the performance of the outbreak detection algorithm in the China infectious disease automated-alert and response system. J. Int. Med. Res..

[19] Zheng J., Huo X., Huai Y., Xiao L., Jiang H., Klena J., Greene C.M., Xing X., Huang J., Liu S. (2016). Epidemiology, seasonality and treatment of hospitalized adults and adolescents with influenza in Jingzhou, China, 2010–2012. PLoS ONE.

[20] Lee H.S., Thiem V.D., Anh D.D., Duong T.N., Lee M., Grace D., Nguyen-Viet H. (2018). Geographical and temporal patterns of rabies post exposure prophylaxis (PEP) incidence in humans in the mekong river delta and southeast central coast regions in Vietnam from 2005 to 2015. PLoS ONE.

[21] Mao Y., Zhang N., Zhu B., Liu J., He R. (2019). A descriptive analysis of the Spatio- temporal distribution of intestinal infectious diseases in China. BMC Infect. Dis..

[22] Kim E.H., Bae J.M. (2018). Seasonality of tuberculosis in the Republic of Korea, 2006–2016. Epidemiol. Health.

[23] Liu Y., Wang X., Liu Y., Sun D., Ding S., Zhang B., Du Z., Xue F. (2013). Detecting spatial-temporal clusters of HFMD from 2007 to 2011 in Shandong Province, China. PLoS ONE.

[24] Zhu B., Liu J., Fu Y., Zhang B., Mao Y. (2018). Spatio-temporal epidemiology of viral hepatitis in China (2003-2015): Implications for prevention and control policies. Int. J. Environ. Res. Public Health.

[25] Ge E., Zhang X., Wang X., Wei X. (2016). Spatial and temporal analysis of tuberculosis in Zhejiang Province, China, 2009–2012. Infect. Dis. Poverty.

[26] Zhu B., Fu Y., Liu J., Mao Y. (2017). Notifiable sexually transmitted infections in China: Epidemiologic trends and spatial changing patterns. Sustainability.

[27] Parra-Amaya M., Puerta-Yepes M., Lizarralde-Bejarano D., Arboleda-Sánchez S. (2016). Early detection for dengue using local indicator of spatial association (LISA) analysis. Diseases.

[28] Xia J., Cai S., Zhang H., Lin W., Fan Y., Qiu J., Sun L., Chang B., Zhang Z., Nie S. (2015). Spatial, temporal, and spatiotemporal analysis of malaria in Hubei Province, China from 2004–2011. Malar. J..

[29] Cao K., Yang K., Wang C., Guo J., Tao L., Liu Q., Gehendra M., Zhang Y., Guo X. (2016). Spatial-temporal epidemiology of tuberculosis in mainland China: An analysis based on Bayesian theory. Int. J. Environ. Res. Public Health.

[30] Guo C., Du Y., Shen S.Q., Lao X.Q., Qian J., Ou C.Q. (2017). Spatiotemporal analysis of tuberculosis incidence and its associated factors in mainland China. Epidemiol. Infect..

[31] Xiang N., Havers F., Chen T., Song Y., Tu W., Li L., Cao Y., Liu B., Zhou L., Meng L. (2013). Use of national pneumonia surveillance to describe influenza A(H7N9) virus epidemiology, China, 2004-2013. Emerg. Infect. Dis..

[32] Engelman D., Steer A.C. (2018). The resurgence of scarlet fever in China Reappraising the cardiosafety of dihydroartemisinin-piperaquine. Lancet Infect. Dis..

[33] You Y., Davies M.R., Protani M., McIntyre L., Walker M.J., Zhang J. (2018). Scarlet fever epidemic in China caused by streptococcus pyogenes serotype M12: Epidemiologic and molecular analysis. EBioMedicine.

[34] Ma C., Gregory C.J., Hao L., Wannemuehler K.A., Su Q., An Z., Quick L., Rodewald L., Ma F., Yan R. (2016). Risk factors for measles infection in 0–7 month old children in China after the 2010 nationwide measles campaign: A multi-site case–control study, 2012–2013. Vaccine.

[35] Zhao Y., Xu S., Wang L., Chin D.P., Wang S., Jiang G., Xia H., Zhou Y., Zhao B., Zhang H. (2012). National Survey of Drug-Resistant Tuberculosis in China. N. Engl. J. Med..

[36] Tang X., Geater A., McNeil E., Deng Q., Dong A., Zhong G. (2017). Spatial, temporal and spatio-temporal clusters of measles incidence at the county level in Guangxi, China during 2004–2014: Flexibly shaped scan statistics. BMC Infect. Dis..

[37] Vencalek O., Kyncl J. (2017). Analysis of the seasonal incidence of acute respiratory infections including influenza (ARI) in the Czech Republic—Possible contribution of the functional data boxplot in epidemiology. Biomed. Pap..

[38] Sun W., Gong J., Zhou J., Zhao Y., Tan J., Ibrahim A.N., Zhou Y. (2015). A spatial, social and environmental study of tuberculosis in China using statistical and GIS technology. Int. J. Environ. Res. Public Health.

[39] Lu S.H., Tian B.C., Kang X.P., Zhang W., Meng X.P., Zhang J.B., Lo S.K. (2009). Public awareness of tuberculosis in China: A national survey of 69253 subjects. Int. J. Tuberc. Lung Dis..

[40] Zou X., Zhou L., Wu H., Chen L., Zhou F., Gong C., Ye J., Ling L. (2019). The role of tuberculosis control institutes in delivering tuberculosis information to domestic migrants in China: A multi-level analysis of a nationwide cross-sectional survey. Int. J. Infect. Dis..

[41] Ben Zakour N.L., Davies M.R., You Y., Chen J.H.K., Forde B.M., Stanton-Cook M., Yang R., Cui Y., Barnett T.C., Venturini C. (2015). Transfer of scarlet fever-associated elements into the group A Streptococcus M1T1 clone. Sci. Rep..

[42] Liao J., Yu S., Yang F., Yang M., Hu Y., Zhang J. (2016). Short-term effects of climatic variables on hand, foot, and mouth disease in Mainland China, 2008–2013: A multilevel spatial poisson regression model accounting for overdispersion. PLoS ONE.

